# Unveiling the N-Terminal Homodimerization of BCL11B by Hybrid Solvent Replica-Exchange Simulations

**DOI:** 10.3390/ijms22073650

**Published:** 2021-03-31

**Authors:** Lukas Schulig, Piotr Grabarczyk, Norman Geist, Martin Delin, Hannes Forkel, Martin Kulke, Mihaela Delcea, Christian A. Schmidt, Andreas Link

**Affiliations:** 1 Department of Pharmaceutical and Medicinal Chemistry, Institute of Pharmacy, University of Greifswald, 17489 Greifswald, Germany; lukas.schulig@uni-greifswald.de; 2 Department of Hematology and Oncology, Internal Medicine C, University Greifswald, 17489 Greifswald, Germany; piotr.grabarczyk@med.uni-greifswald.de (P.G.); martin.delin@med.uni-greifswald.de (M.D.); hannes.forkel@med.uni-greifswald.de (H.F.); christian.schmidt@med.uni-greifswald.de (C.A.S.); 3 Department of Biophysical Chemistry, Institute of Biochemistry, University of Greifswald, 17489 Greifswald, Germany; norman.geist@uni-greifswald.de (N.G.); martin.kulke@uni-greifswald.de (M.K.); delceam@uni-greifswald.de (M.D.)

**Keywords:** BCL11B, CCHC zinc finger, homodimerization, protein folding, protein-protein docking, replica-exchange molecular dynamics, TIGER2hs, TIGER2h

## Abstract

Transcription factors play a crucial role in regulating biological processes such as cell growth, differentiation, organ development and cellular signaling. Within this group, proteins equipped with zinc finger motifs (ZFs) represent the largest family of sequence-specific DNA-binding transcription regulators. Numerous studies have proven the fundamental role of BCL11B for a variety of tissues and organs such as central nervous system, T cells, skin, teeth, and mammary glands. In a previous work we identified a novel atypical zinc finger domain (CCHC-ZF) which serves as a dimerization interface of BCL11B. This domain and formation of the dimer were shown to be critically important for efficient regulation of the BCL11B target genes and could therefore represent a promising target for novel drug therapies. Here, we report the structural basis for BCL11B–BCL11B interaction mediated by the N-terminal ZF domain. By combining structure prediction algorithms, enhanced sampling molecular dynamics and fluorescence resonance energy transfer (FRET) approaches, we identified amino acid residues indispensable for the formation of the single ZF domain and directly involved in forming the dimer interface. These findings not only provide deep insight into how BCL11B acquires its active structure but also represent an important step towards rational design or selection of potential inhibitors.

## 1. Introduction

### 1.1. Role of BCL11B

The BCL11B gene encodes a Krüppel-like, sequence-specific zinc finger (ZF) transcription factor that acts predominantly as a repressor. It executes its function via interactions with various chromatin modifying proteins and complexes leading to creation of a transcriptionally inactive environment. The catalogue of the BCL11B interacting proteins grows continuously and includes both Zn^2+^- as well as NAD^+^-dependent histone deacylases (HDACs, sirtuins), HP1α, methyltransferases (SUV39H1) or retinoblastoma associating proteins (RBBP4 and RBBP7), among others [1,2,3,4,5,6]. Despite the repressive nature of most BCL11B-containing complexes, activation of certain signaling pathways such as mitogen-activated protein kinases (MAPK) or phosphoinositide-3 kinases (PI3K) may lead to initiation of a composite sequence of posttranslational modification and conversion of BCL11B from a repressor into a transcription activator at the same regulatory elements [7,8,9].

The expression of BCL11B has been detected in a variety of tissues and its importance for their normal development and function was established by various knockout models in mice. BCL11B loss during embryogenesis results in an incorrect structure and function of various organs and tissues of the central nervous system, skin, mammary glands and lymphoid compartment [10,11,12,13,14,15]. The relevance of the gene in humans was recently confirmed by a discovery of the first germline *de novo* mutations in BCL11B locus [16]. The single heterozygous substitution (N441K) in the second CCHH zinc finger domain led to a variety of abnormalities, including severe immunodeficiency resulting from disrupted hematopoietic stem cell migration and arrested T-lineage development. Further developmental defects were observed in skin, bones and neuronal tissue accompanied by mental retardation.

In contrast to severe and frequently lethal abnormalities observed in constitutive BCL11B knockout models, tamoxifen-inducible, CRE-lox mediated excision of the floxed Bcl11b locus in adult animals resulted in mild but intriguing outcome [17]. The lack of BCL11B expression appeared not to have any detrimental effect in most tissues except for the lymphatic system. Here, BCL11B ablation caused the reprogramming of T lymphocytes into cells functionally resembling normal cytokine activated NK cells (iTNKs). Remarkably, iTNK cells demonstrated superior proliferative capacity and potently eliminated tumor cells in vitro and in vivo. Provided the findings can be reproduced in human T lymphocytes, iTNK cells might represent a potential source for cellular therapies against cancer.

The vital role of BCL11B in developmental processes makes alterations of this gene responsible for a variety of pathologies. Interestingly, both loss of function mutations as well as elevated expression were reported to be associated with disease development, even within one disease entity. It has been reported that approximately 10% to 16% of T cell acute lymphoblastic leukemia (T-ALL) cases carry mutations altering the DNA-binding properties of BCL11B [18]. Along with the similar findings in mouse γ-radiation induced T cell lymphomas [19,20], the recurrent genetic lesions de-activating BCL11B function strongly indicate a tumor suppressor function of the gene. However, the majority of T-ALL and other malignancies originating from BCL11B-positive tissues, such as head and neck squamous cell carcinomas (HNSCC), Ewing sarcomas or neuroblastomas (NBs) are characterized by elevated expression of non-altered gene ([21,22], unpublished observation). Moreover, we and others showed that high BCL11B levels are critical for prevention of apoptosis and accumulation of DNA damage in malignant T-cells [23,24,25,26]. This observation could be extended to other tumor types including HNSCC and NB.

Previously, we reported the identification of a novel atypical zinc finger domain (CCHC-ZF) functioning as a dimerization interface of BCL11B. This atypical zinc finger domain and its dimer formation were shown to be critically important for efficient repression of the target genes and BCL11B function [27].

### 1.2. Enhanced Molecular Dynamics Simulations

Ab initio simulations of protein folding and aggregation can be realized by modern enhanced sampling methods and have proven to be a useful tool for structure prediction. One of the most common methods describes temperature replica-exchange simulations (T-REMD), also known as parallel tempering. Multiple copies of the system are simulated simultaneously at different temperatures and frequently attempt to exchange their temperature based on the Metropolis sampling criterion (MSC). The simulation on higher temperatures increases the kinetic energy, which effectively reduces energy barriers between states and enhances the transition times between conformations in local minima. Connecting the state distributions at different temperatures by the MSC results in a guided random walk in the potential energy space for replicas and a Boltzmann distribution of states on all temperatures.

T-REMD simulations are considered accurate [28,29] and reliable if the system is simulated under valid conditions, i.e., explicit representation of solvent. With largely increased degrees of freedom due to the solvent molecules, the number of replicas required for sufficient exchange rates in T-REMD skyrockets quickly with increasing size of the solute. As a result, computational cost becomes the major downside of T-REMD simulations, even for small biomolecular systems.

In the past, different variations of replica-based algorithms were developed to tackle this problem. One of them is Temperature Intervals with Global Exchange of Replicas (TIGER2) combined with a hybrid solvent scheme (TIGER2hs) [30,31]. It closely resembles the T-REMD approach, with two important exceptions: Prior to the exchange attempts, all replicas are cooled down to the baseline temperature in TIGER2hs. Exchanges are attempted between the current baseline replica and the other replicas in ascending order of their position on the temperature ladder. In contrast, exchanges in REMD are attempted only for neighboring replicas in temperature space. Secondly, in TIGER2hs the bulk water is replaced by a continuum model (e.g., Generalized Born) and only the first two water shells and the solute atoms are included for the potential energy calculations that drive the exchange decisions. By removing noise in the potential energy arising from fluctuations in the bulk water, the number of replicas is drastically reduced. Additionally, by accepting multiple conformations to the baseline ensemble of states at each cycle, a significant reduction in convergence time can be achieved. As the solute is only embedded into an implicit solvation for the exchange decisions, the accuracy of the sampling with explicit solvation is retained.

### 1.3. Zinc Finger Proteins in Force Field-Based Simulations

Metal ions are crucial for the structure and function of around 30% of all proteins found in humans. Unfortunately, the nature of coordinative bonds makes it a challenging task to correctly represent them in molecular mechanical simulations. Especially for protein folding simulations, binding to the metal ion includes deprotonation, charge transfer and polarization of ligand residues. Such effects require dynamic topology and charge adjustments, which are typically not available in simulations with classical protein force fields. Parameters for metal ions can be roughly divided into bonded and nonbonded ones, where the simplest is a single point charge particle as often used for monovalent ions. Sakharov and Lim proposed a method for zinc finger proteins, where the charge transfer effect is calculated depending on a linear function of distance *r* between metal and coordinating ligand atom [32]. Based on that scheme, we also extended NAMD version 2.13 to support single charge adjustments within Tcl scripts and performed multiple simulations, using the equation variables from Li et al. but all attempts remained unsuccessful (data not shown) [33].

Chang et al. investigated possible pathways of the folding of zinc finger proteins. They used a method that could be described as molecular dynamics simulations of the unfolding process. Chang et al. came to the conclusion that the CXXC motif and the corresponding β-sheet fold first, and afterwards become stabilized by zinc ion binding [34]. This is consistent with the CXXC loop folding mechanism, which is guided by backbone hydrogen bond interactions with the cysteine thiol groups [35]. We also attempted ab initio protein folding simulations with different lengths of the β-sheet forming residues with and without zinc ion (data not shown). The β-fold could only be observed without metal ion but no defined minimum structure could be obtained, supporting the assumption of a distinct order for each individual folding step of the overall structure that could not be achieved with the applied charge transfer scheme.

To overcome these issues, we performed a multi-stage structure prediction with multiple in silico methods. By first predicting the core zinc finger structure via homology modeling, followed by an optimization step, we were able to use a bonded model for the zinc complex during the subsequent enhanced sampling MDS (molecular dynamics simulations). Sampling only a local conformational space around an initial homology model, as well as the optimization of dimer models were earlier successfully demonstrated with TIGER2hs [36].

## 2. Results and Discussion

### 2.1. Homology and Loop Modeling

**Figure 1:**
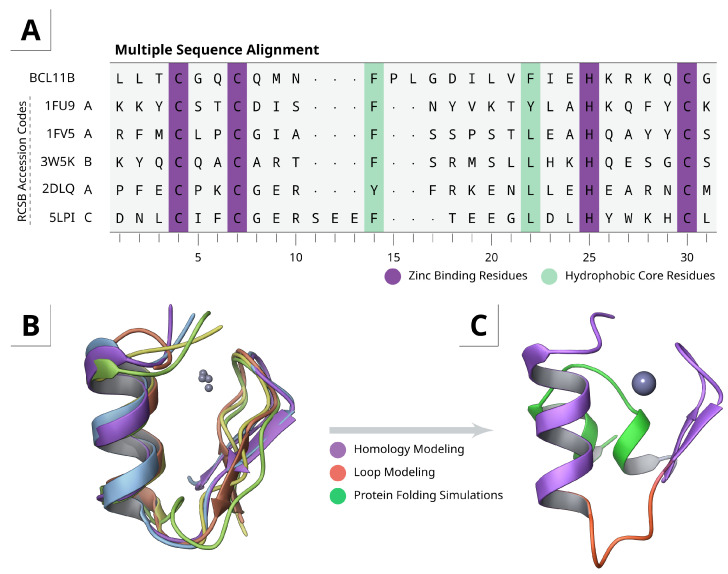
(**A**) Multiple sequence alignment of known CCHC-ZF with important residues highlighted in purple and light green. (**B**) Structural alignment shows a high degree of similarity and the classical ββα-fold for all of them. (**C**) Final model after homology modeling, loop refinement (orange) and replica-based protein folding simulations (green).

To overcome the aforementioned problems with metal ions in force field-based simulations, the initial structure was generated using a two-stage homology modeling and refinement approach. Due to the rather unique sequence and atypical length of 14 amino acids between C61 and H76, no high-quality templates were available. Structural alignment of other known CCHC zinc fingers unveiled common motifs (Figure 1A,B). A β-sheet, starting from the N-terminal side, containing the first two zinc binding cysteines, followed by a variable length loop and a short α-helix. The stabilizing hydrophobic core is formed by at least two hydrophobic residues after coordination of histidine to the zinc ion. The overall ββα fold is therefore similar to classical DNA-binding zinc fingers [37].

Since the tetrahedral geometry of the zinc complex is rather fixed, the orientation between the β-sheet and α-helix is constrained. Both structural motifs were therefore used for homology modeling separately and reconnected afterwards through de novo linker/loop modeling (Figure 1C), resulting in a more coherent model than full size homology modeling only.

Beside the hydrophobic core formed by F65 and F73, residue K77 is also crucial for stability. It is known that adjacent positively charged residues facilitate thiol ionization by decreasing the $pK_{a}$ value [35], which could also be confirmed by K79A mutants (Section 2.4). Binding of histidine to the zinc ion results in a polarization of the imidazole ring and is possibly enhanced by a hydrogen bond to the Q80 sidechain amide which requires an α-helical fold. Although this interaction affects the metal complex [38], Q80A mutants have proven that it plays a subordinate role.

The CXXC motif typically forms a β-turn, even in metal-free proteins, due to hydrogen bond interactions with backbone carbonyl oxygen atoms. A similar fold of the CGQC motif from BCL11B can be found in cereblon isoform 4 from *Magnetospirillum gryphiswaldense* (PDB: 4V2Y). Altogether the structural alignments and secondary interactions are a strong indication for the ββα-fold.

### 2.2. Model Extension and Monomer Conformational Sampling

Although the ββα-fold can be predicted from other known CCHC zinc finger proteins, we assumed that the short hydrophobic SGLGLMVGG motif on the N-terminal side could round off the overall structure and stabilize a putative dimerization interface (Figure 1C). Since this region is often flexible and typically not resolved in NMR or crystal structures, no suitable templates are available at this time. The structure obtained from the homology and loop modeling, was extended by simply adding these additional N-terminal residues as disordered coil.

**Figure 2:**
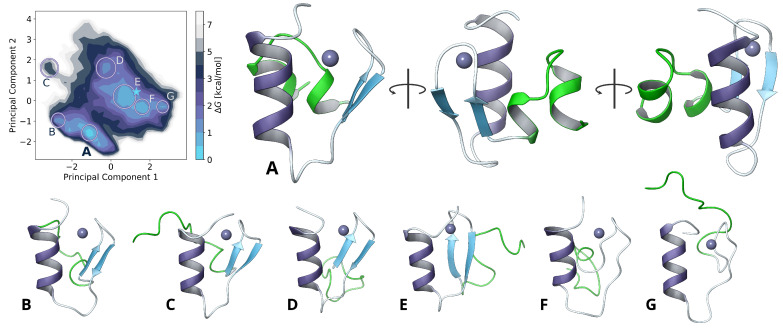
(top left) Free energy landscape obtained from TIGER2hs sampling and dihedral principal component analysis with global minimum (**A**) from different angles and other common structures (**B**–**G**) with different loop conformations colored in green. The initial homology modeled structure is highlighted by a blue star on the heatmap.

To resolve this N-terminal structure and to refine the overall homology modeled core structure, we sampled nearly one microsecond of simulation time using the TIGER2hs replica-exchange algorithm. Weak secondary structure restraints were applied as harmonic potentials on hydrogen bonds for the α- and β-folded regions to preserve the previously determined core structure of the zinc finger motif. A reduction of the conformational degrees of freedom at the core hydrophobic region also avert insurmountable energy barriers from large structural changes, trapped by zinc coordination in the applied bonded model. Nevertheless, all residue sidechains were left flexible to allow optimizing the structure of the previous step. This facilitated rearrangements in the hydrophic core and the linking loop region in explicit solvation (Figure 2) to obtain further insights of the F65 and L67 orientation and possible contribution to dimerization.

**Figure 3:**
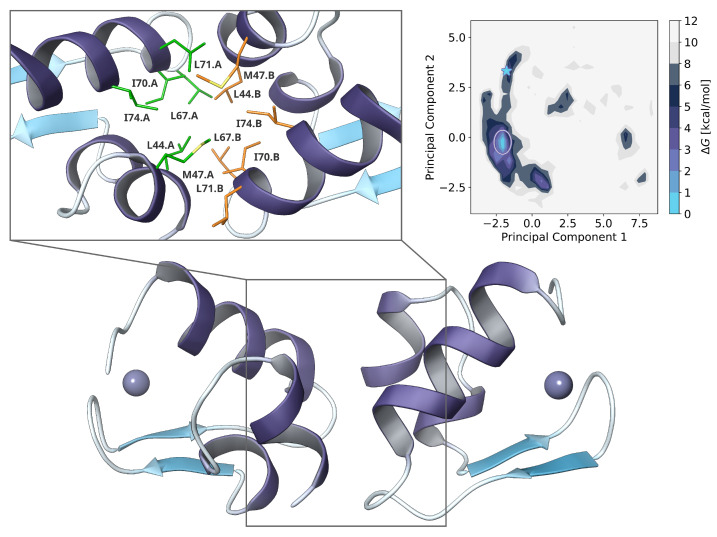
(**right**) Free energy landscape obtained from principal component analysis of pairwise atom-atom distances from TIGER2h ensemble. The starting structure is highlighted by a blue star on the heatmap, while a white circle highlights the global minimum. (**bottom**) Global minimum dimer configuration. (**left**) The magnified region depicts the binding interface of both monomers with their corresponding residues colored in green and orange, respectively.

The added N-terminal sequence revealed two short helical motifs, separated by a flexible GGP coil. Both are stabilized by hydrogen bonds to D54, R78 and ionic interactions of D52 and K77. Additional interactions with S84 could be observed during the simulation. The hydrophobic core is enclosed by L44, M47, V48 and P53, while shaping a distinct hydrophobic surface on the bottom side (Figure 3).

### 2.3. Dimer Protein-Protein-Docking and Refinement

The protein-protein-docking algorithm as implemented in Molecular Operating Environment (MOE) uses the popular fast Fourier transform approach (FFT docking) with coarse-grained representation of residues. It allows an exhaustive search of the docking space with low computational efforts [39]. However, major drawbacks of the FFT docking are that protein flexibility and solvation effects are only taken into account to a limited extent. Especially for small, highly dynamic proteins such as this zinc finger motif, near-native structures are hardly obtained. A combination with molecular dynamics simulations can solve this issue but depends on the preselection of docking poses and they can be easily trapped in local minima.

Hence, we rely on a different approach here and the FFT docking only aimed to provide a good starting structure for the subsequent replica-exchange MDS with TIGER2h, as this may reduce the sampling time of the protein complex formation in solution. Only a low number of replicas were necessary to efficiently explore the dimer interactions. The maximum temperature of 450 K was sufficient and could be probably further reduced for fine controlled diffusion on the temperature space, similar to an increased number of replicas in T-REMD. Backbone dihedral restraints allowed rigid body-like sampling with flexible sidechains, to allow dimer pose explorations with simultaneous local structure optimizations and adaptations.

The initial pose from FFT docking was selected by visual inspection with focus on symmetry. Both monomers had some kind of linear orientation to each other and main interactions at the hydrophobic side of L44, L67 and I70. Since this was only performed to improve the starting conditions for the replica-exchange simulation, only a single pose was selected.

After around 90 ns of TIGER2h simulation, a distinct minimum could be obtained with an energy barrier of >$2.5k\mathrm{cal}/{\mathrm{mol}}^{-1}$ on the free energy landscape. The orientation had changed nearly to a right angle between both α-helical structures, while increasing the total interface surface and remaining highly symmetrical. Further hydrophobic interactions were now formed also by M47, L71 and I74.

We also performed TIGER2h simulations on larger oligomeric complexes such as tetramers (data not shown) but no apparent conformation was found so far. It is still not clear if these also exist and should be investigated in future studies.

### 2.4. Residue Importance by FACS-FRET Measurements

**Figure 4:**
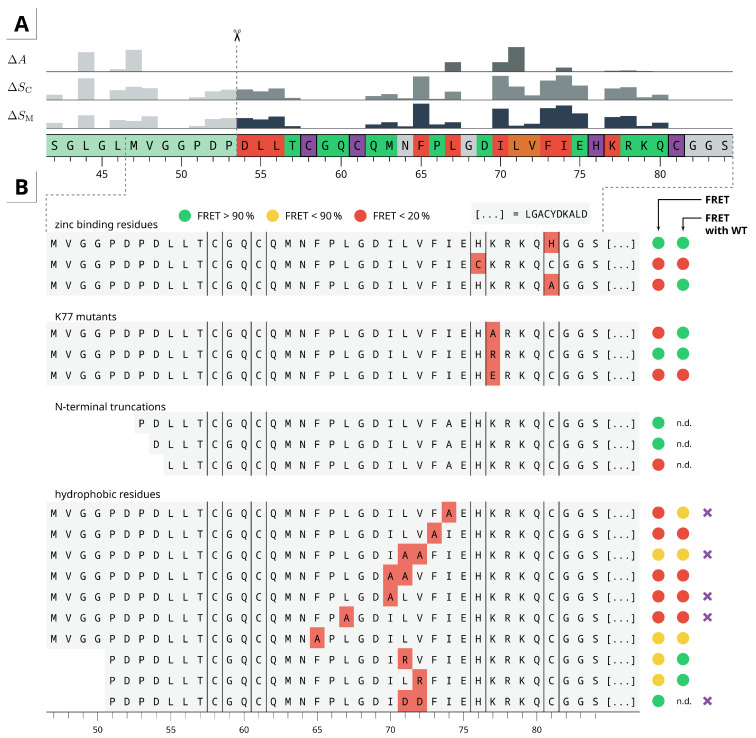
(**A**) (bottom) Summary of all experimental residue mutations colored by their effect on dimerization (red/orange: mutations prevent dimerization, green: mutation to alanine does not affect dimerization, purple: metal-binding residues). The bar charts (top) are the normalized changes in energy from in silico alanine scanning for monomer ($\Delta S_{M}$) and complex ($\Delta S_{C}$) stability, as well as affinity (ΔA). Higher values mean a negative effect on both parameters. (**B**) Excerpt of key mutations and their dimerization potential to itself and wild-type. Purple crosses denote selected mutations for further protein-protein docking simulation with TIGER2h. (n.d.: not determined). More information can be found in Supplementary Materials.

To get further insights on how individual residues affect the zinc finger dimerization, a wide range of mutants were constructed and experimentally screened via FACS-FRET assay. Figure 4A shows a summary and the corresponding in silico alanine scanning results obtained from our model. Of the zinc binding residues only H76 and C81 were mutated. Although C81H probably just resembles a CCHH type ZF, C81A shows dimerization only with WT. A partial disruption of the metal complex may result in a ZF-like motif with intact α-helix, where most of the interacting residues are located. This could be sufficient to form a stable protein-protein complex, especially if the lack of ligands is compensated by adjacent residues. Similar effects appear when mutating K77 to alanine. It has been reported by Kluska and others that thiol ionization (deprotonation), can be promoted by neighboring positively charged groups. Thus, the positively charged lysine sidechain is critical for thiolate formation due to inductive effects, resulting in decreased $pK_{a}$ value of the thiol group. For CCCC constructs such as H76C, the number of protonated cysteine residues is increased at physiological pH [35], which disfavors metal coordination.

Of the hydrophobic core residues F65 and F73, dimerization was only observed for F65A constructs, presumably due to the atypical loop length and compensating effects with other residues such as L67. The residues D54, L55 and L56 are stabilizing the β-fold, the hydrophobic core and the interaction interface in our predicted model. Mutation of these residues to alanine therefore negatively affects folding and binding. L67A, I70A and I70A mutants all abolish dimerization with itself or wild-type and hence they seem to be more exposed, we expect them to be directly involved in binding. This hypothesis is also supported by mutations of M63, L71 and V72 to alanine, as well as positively and negatively charged residues. For L71A, the in silico alanine scanning predicted the largest change in affinity (ΔA) and a medium change in complex stability ($\Delta S_{C}$), which seems rather overestimated from a structural point of view, since it is at the outer edge of the binding interface.

The charged residues E75, R78 and K79 might enhance the α-helix folding or stability but they are not important for the dimerization as observed by several mutants. Therefore, it seems reasonable to suppose that the binding is directed by hydrophobic interactions only.

Our predicted model revealed residues such as L44 or M47 that might be directly involved in the binding interface. We therefore truncated the protein at the N-terminal side to the shortest possible length and observed that the ββα-fold is still sufficient for dimerization.

### 2.5. Dimer Protein-Protein-Interactions of Mutants

The results from experimental mutations pointed out the crucial residues for dimerization. However, besides the metal-binding residues, the positively charged K77 and the hydrophobic core forming F65 and F73, it is not clear whether the dimerization itself or just the monomer folding is affected. Therefore, we selected a few variants for additional replica-exchange simulations. Among them, especially those where residues found to be directly involved in interaction and no FRET signal was observed. To avoid new expensive folding simulations, the shortest possible construct that still shows dimerization in the FACS-FRET assay was used (starting from D54). From our predictions, this only includes the ββα-fold without any adjacent residues. Leaving the backbone of the first two N-terminal residues unrestrained still allowed a slight flexibility.

Since the TIGER2h simulations have proven to exhaustively explore the dimer configuration space, no inital FFT docking was performed for the mutants. Instead, we simply truncated the final model obtained from the prior sampling at the N-terminal region and mutated the amino acids. A total of five mutants (highlighted in Figure 4B) and the short wild-type construct were selected. The latter was used as reference for comparison since some of the predicted interacting residues of the full-length zinc finger were missing now.

**Figure 5:**
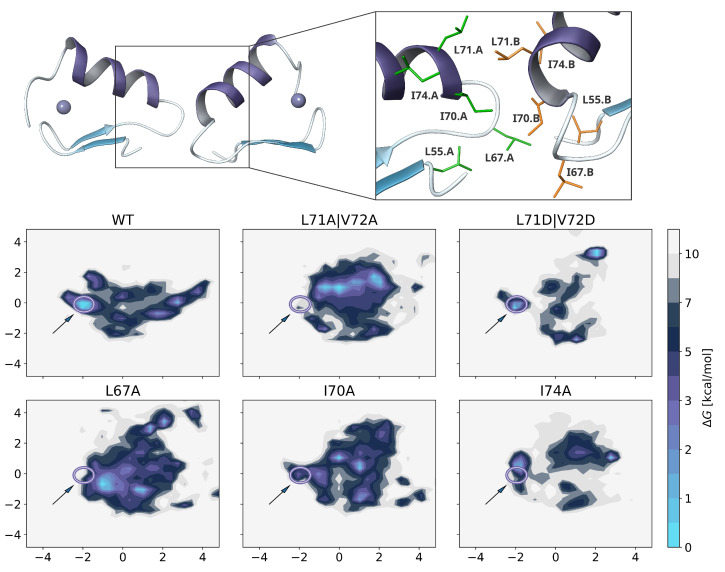
Free energy landscapes obtained from TIGER2h protein-protein docking simulations for selected mutants, marked in Figure 4. The circle highlights the minimum wild-type structure based on pairwise backbone atom distance PCA, and is shown above. Similar structures appear at similar spots on the conformational landscape, due to injection of the WT principal axes for also the mutant constructs. Interface residues and orientation of the truncated wild-type conformation is similar to the full-length zinc finger protein (Figure 3) and was used as reference.

As shown in Figure 5 a clear minimum complex structure is preserved for the truncated wild-type construct, even though it is less stable than the full-length zinc finger protein. The orientation and binding interface of both monomers is similar, while residues such as I70 and I74, which were important for stabilizing the interface in our predicted model, are now directly involved in binding. Main interactions are driven by the hydrophobic residues L67, I70, L71, I74 and at least to some extent L55 and F73. The experimental mutations also have substantiated their importance for dimerization (Figure 4).

Although for L71A|V72A only a medium FRET signal was observed, L71D|V72D mutations apparently does not affect the dimerization and a wild-type-like binding mode2 could be obtained to a significant extent. The V72D residue does not participate in any direct interactions and is simply solvent exposed but L71D is bridged to itself via water molecules or sodium ions, which stabilizes the protein-protein interface. For the remaining mutants, the WT-like conformation was sometimes found in the ensemble with variable proportions, but they were not stable enough to result in a meaningful minimum structure with distinct energy barriers. Hence, the calculations are in a good agreement with the experimental FRET results.

## 3. Conclusions and Perspectives

In this study, we identified the molecular basis for homodimerization of the N-terminal CCHC zinc finger domain of BCL11B, which is mainly driven by the hydrophobic residues L67, I70 and I74. The combination of homology modeling and replica-based protein folding simulations, unveiled a classical ββα-fold for the core region and small adjacent helical structures that build up and stabilize a distinct binding interface, which can be targeted by small molecule inhibitors or different approaches such as stapled peptides. The unique sequence and association of BCL11B with a wide range of diseases, makes it a promising target for novel drug therapies.

The identification of individual residue contributions via FACS-FRET assay was a useful tool to verify the computational models and to obtain further insights on the tertiary and quaternary structure of the CCHC zinc finger motif. Subsequent simulations of various mutants could be specifically selected and are in a good agreement with the experimental data.

To our knowledge, this is the first attempt to apply the hybrid solvent replica-exchange algorithm TIGER2h for protein-protein docking. Our results show significant advantages compared to the classical FFT docking approach, with manageable computational costs. Using this method, we were able to extensively sample a vast number of possible states in explicit solvation, without dependence on coarse-grained representations or scoring functions. Fine-grained and adaptive control of restraints allow flexibility to any desired extent. Future studies should investigate the application and scalability on other protein complexes.

## 4. Methods

### 4.1. Homology and Loop Modeling

All calculations were performed using the Molecular Operating Environment (MOE) version 2019.01. The NMR structure of the ninth FOG-type CCHC zinc finger from Ush (zinc finger protein U-shaped) was taken from the RCSB database (1FU9) [40] and prepared using *QuickPrep*. The target sequence of BCL11B, ranging from L55 to S84, was aligned on the template sequence, with all metal-coordinating residues constrained. A total of three main chain models were calculated at a temperature of 300 K, with each having ten sidechain models. The final homology model was minimized after applying AMBER ff14SB parameters [41].

To optimize the loop region between the α-helix and β-sheet (F76-I70), all residues were removed and reconnected again by *linker modeling*. The resulting structure was then optimized through *loop modeling* using a de novo search and knowledge-based approach. Overall, 10,000 loop conformations were generated and sorted according to their score value. The top 15 conformations were visually inspected and submitted to sidechain prediction and minimization. Since the resulting structures were largely equal, only the top scoring conformation was kept for further simulations.

### 4.2. Simulation System Preparation

The structure obtained from loop modeling was further extended by adding 13 residues at the N-terminus (SGLGLMVGGPDPD) and capping groups (acetyl, *N*-methyl-amide) on both ends, respectively. Protonation was refined by *Protonated3D* (MOE 2019.01), followed by a constrained minimization to remove any steric clashes. AMBER ff14SB and Zinc AMBER Force Field (ZAFF) parameters [42] were set by tLeap (AmberTools 16) [43]. The total system charge was neutralized by adding appropriate amounts of sodium ions. Water molecules, represented as TIP3P model, were added to a cubic simulation cell with side lengths of 6 nm. Hydrogen mass repartitioning was applied by ParmEd 3.1.0 to enable a 4 fs timestep [44].

### 4.3. General Molecular Dynamics Setup

The molecular dynamics simulations were performed using NAMD 2.13 [45] with the same settings throughout all simulations. Short-range interaction cutoffs for van der Waals (vdW) and Coulomb interactions were set to 1.0 nm with 0.1 nm switching functions, while long-range electrostatic interactions were described by the particle mesh Ewald method (PME). Bond lengths to hydrogen atoms were constraint by the RATTLE algorithm. Temperature and pressure were controlled by Langevin thermostat with 1 p$s^{-1}$ damping and Langevin piston barostat at 100 fs period and 200 fs decay time.

### 4.4. Monomer Conformational Sampling

Folding simulations were based on the TIGER2hs enhanced sampling method and implemented using the genuine code from reference [31].

Prior to the replica-exchange simulation, the hydration shell size for the hybrid solvent energy evaluation was determined. After an initial minimization of 50,000 steps, the system was equilibrated for 400 ps NVT and NPT, each. During the production phase of 10 ns, snapshots were collected every 2 ps. The respective radial distribution function for water oxygen atom to their nearest solute atom was evaluated in bins of 0.005 nm, according to the procedure presented in the original article. The number of water molecules can then be defined as function over the distance *r* to the solute. With r= 0.41nm, N=350 water molecules were obtained.

For the TIGER2hs simulation, 64 replicas were used on an exponential temperature scale ranging from 280 K to 600 K, including one replica for the hybrid solvent energy calculation. Each sampling cycle consisted of 20 ps sampling time, followed by 10 ps rapid quenching, cooling down all replica to the baseline temperature prior to each exchange attempt. Residues forming the initial ββα-fold were restrained by additional harmonic potentials on their backbone hydrogen bonds. Thus, only a local conformational space is sampled during the TIGER2hs simulation and no significant change in the shell size is expected. Otherwise, for solutes of this size, potential biases towards folded or unfolded states can occur, when the shell size significantly varies between states, during the sampling. Exchanges are driven by energies from hybrid explicit/implicit solvation and used the Generalized Born model with solvent accessible surface area term (GBSA) as implemented in NAMD, using default parameters [46]. No periodic boundary conditions were applied at this step.

A total of 150,000 conformations were collected during a sampling time of 900 ns and analyzed using dihedral principal component analysis (dPCA). By computing the two-dimensional histogram of the first two principal components, at a resolution of 30×30 bins, probabilities were calculated as Gibbs free energies ΔG using the Boltzmann inversion to obtain the folding free energy landscape.

### 4.5. Dimer Protein-Protein-Docking

An initial complex was obtained using the rigid body protein-protein-docking algorithm in MOE 2019.01 by simply using one monomer as target and ligand protein, respectively. System preparation for NAMD was then performed as described above, except for a cubic simulation cell with side lengths of 8 nm. Since binding and unbinding events result in large deviation of the hydration shell size, the TIGER2hs algorithm is currently not applicable on such multi-molecule systems. Therefore, only the TIGER2h scheme without explicit water shells was applied [30]. Temperatures were again distributed exponentially from 280 K to 450 K on 11 replicas, with one additional replica doing the hybrid energy calculations. To implement a rigid body fashion during the sampling, harmonic restraints to all backbone dihedral angles, excepted for the first and last two residues, were put in place, at a force constant of 25 kcal${\mathrm{mol}}^{-1}$$\deg^{-2}$. Around 11,000 baseline snapshots were collected during a total simulation time of 90 ns.

To obtain the free energy landscape for the dimer configurations, we calculated the normalized pairwise distances of all heavy atoms between monomer A and monomer B, followed by dimension reduction with principal component analysis. Calculation of the free energy surface from the two major principal components was carried out as previously for the dPCA.

### 4.6. Mutants Protein-Protein-Docking

The same procedure as for the full-length zinc finger protein was applied to all mutants, except that the initial pose was taken from the dimer configuration obtained by TIGER2h simulation instead of FFT docking. The shortest possible construct (D54 to S84) was used, and residues were mutated accordingly. All simulations were performed for at least 90 ns, resulting in more than 10,000 baseline snapshots each. The dimer configurations were analyzed as previously for the full-length system. By inducing the principal axes of the shortened wild-type simulation, the mutant states are drawn on the same conformational landscape and it can be easily appreciated if similar minimum structures are obtained.

### 4.7. Alanine Scanning

All calculations were performed with Maestro 2020.4 and OPLS3e force field parameter [47]. A single snapshot was taken from the free energy minimum region of the TIGER2h protein-protein-docking simulation. The complex was submitted to a restrained minimization to remove remaining kinetic energy after deleting all water molecules and counter ions. Residue scanning was performed twice. In the first run, only residue contributions (ΔStability) in a monomer structure were obtained by mutating the residues to alanine. For the second run, the full complex was mutated symmetrically on both monomers, to preserve a homodimeric complex and ΔStability and ΔAffinity were estimated simultaneously. Glycine residues were not mutated to avoid false positive results due to steric clashes and metal-binding residues were omitted.

### 4.8. FACS-FRET Assay

To enable detection of the CCHC zinc finger interactions inside the cells, we adopted the FACS-based fluorescence resonance energy transfer approach described elsewhere [27]. In brief, the BCL11B fragments to be checked for their direct reciprocal interaction were cloned into the plasmids encoding the classical FRET pair ECFP and EYFP. The fragments corresponding to the wild-type BCL11B CCHC zinc finger and its various mutants were synthesized as double stranded DNA oligonucleotides and contained at least 15 base pair overlap with the vector sequences surrounding BglII restriction site (Integrated DNA Technologies, Leuven, Belgium). The synthetic cDNA were cloned into BglII-digested plasmids downstream of ECFP and EYFP using the ligase-free cloning system (Takara Bio Europe, France). The sequence fidelity and the presence of the in-frame ECFP- and EYFP-CCHC fusions were verified by Sanger sequencing (LGC Genomics GmbH, Germany). The positively verified vectors were produced using endotoxin-free plasmid isolation procedures (HiPure Plasmid Maxiprep Kit, Thermo Fisher Scientific, Lithuania).

To validate the occurrence of a putative interaction, the candidate BCL11B fragments-encoding ECFP and EYFP plasmids were transfected into HEK293 cells growing in standard conditions with calcium-phosphate gene transfer procedure (CalPhos mammalian transfection kit; TaKaRa Bio Europe, France).

After 24 h of incubation, transfected cells were detached from the culture vessels with Accutase cell dissociation reagent (Thermo Fisher Scientific) and the fluorescent signals were measured by means of a Navios flow cytometer (Beckman Coulter GmbH, Germany). To detect the expression of the putative interaction partners, the analyzed cells were excited with two different wavelengths. The FRET donor (ECFP) was excited with violet laser (405 nm) and detected in fluorescence channel defined by 450/50 nm bandpass optical filter. The FRET acceptor signal (EYFP) was collected employing the 575/30 nm filter upon excitation by the blue laser (488 nm). The FRET signal indicating the close proximity of ECFP and EYFP fusions (less than 10 nm) was detected as an elevated fluorescence collected at 540/40 nm channel. The enhanced fluorescence at this wavelength results from added excitation (475 nm) originating from violet laser-excited ECFP. The background FRET fluorescence levels were established by transfecting non-fused and non-interacting ECFP- and EYFP-encoding vectors. Vector encoding ECFP-EYFP fusion protein served as a FRET positive control. Each BCL11B fragment tested for the dimerization capabilities was transfected at different ECFP-to-EYFP plasmid ratios and at least 5 replicates were performed for each condition.

## Data Availability

Not applicable.

## Supplementary Materials

The following are available at https://www.mdpi.com/article/10.3390/ijms22073650/s1, Figure S1: Full overview of all experimental residue mutations and their dimerization potential to itself and wild type.; Figure S2: Relative probability of residue contacts during the TIGER2h protein-protein docking simulations for each mutant protein.; Video S1: Dimer docking.

## Abbreviations

The following abbreviations are used in this manuscript:

ECFP	Enhanced Cyan Fluorescent Protein.
EYFP	Enhanced Yellow Fluorescent Protein
FACS	Fluorescence-activated Cell Sorting
FFT	Fast Fourier Transform
FRET	Fluorescence Resonance Energy Transfer
GBSA	Generalized Born Model with Accessible Surface Area Term
(d)PCA	(Dihedral) Princicipal Component Analysis
MDS	Molecular Dynamics Simulation
REMD	Replica-Exchange Molecular Dynamics
TIGER2h(s)	Temperature Intervals with Global Exchange of Replica with Hybrid Solvent (and Shell)
ZF	Zinc Finger
