# Supplementary material for: Unveiling the N-Terminal Homodimerization of BCL11B by Hybrid Solvent Replica-Exchange Simulations
Source: Int J Mol Sci. 2021 Mar 31;22(7):3650. doi: 10.3390/ijms22073650 (PMC8036541; doi:10.3390/ijms22073650)
Supplement: Supplementary file 1 [file ijms-22-03650-s001.zip › LS_BCL11B_ZF_SI.pdf]

SUPPORTING INFORMATION

Unveiling the N-Terminal Homodimerization of BCL11B by Hybrid Solvent Replica-Exchange Simulations

Lukas Schulig [1,†], Piotr Grabarczyk [2,†], Norman Geist [3], Martin Delin [2], Hannes Forkel [2], Martin Kulke [3], Mihaela Delcea [3], Christian A. Schmidt [2] and Andreas Link [1,*]

[1] Department of Pharmaceutical and Medicinal Chemistry, Institute of Pharmacy, University of Greifswald, Greifswald, 17489, Germany

[2] Department of Hematology and Oncology, Internal Medicine C, University Greifswald, Greifswald, 17489, Germany

[3] Department of Biophysical Chemistry, Institute of Biochemistry, University of Greifswald, Greifswald, 17489, Germany

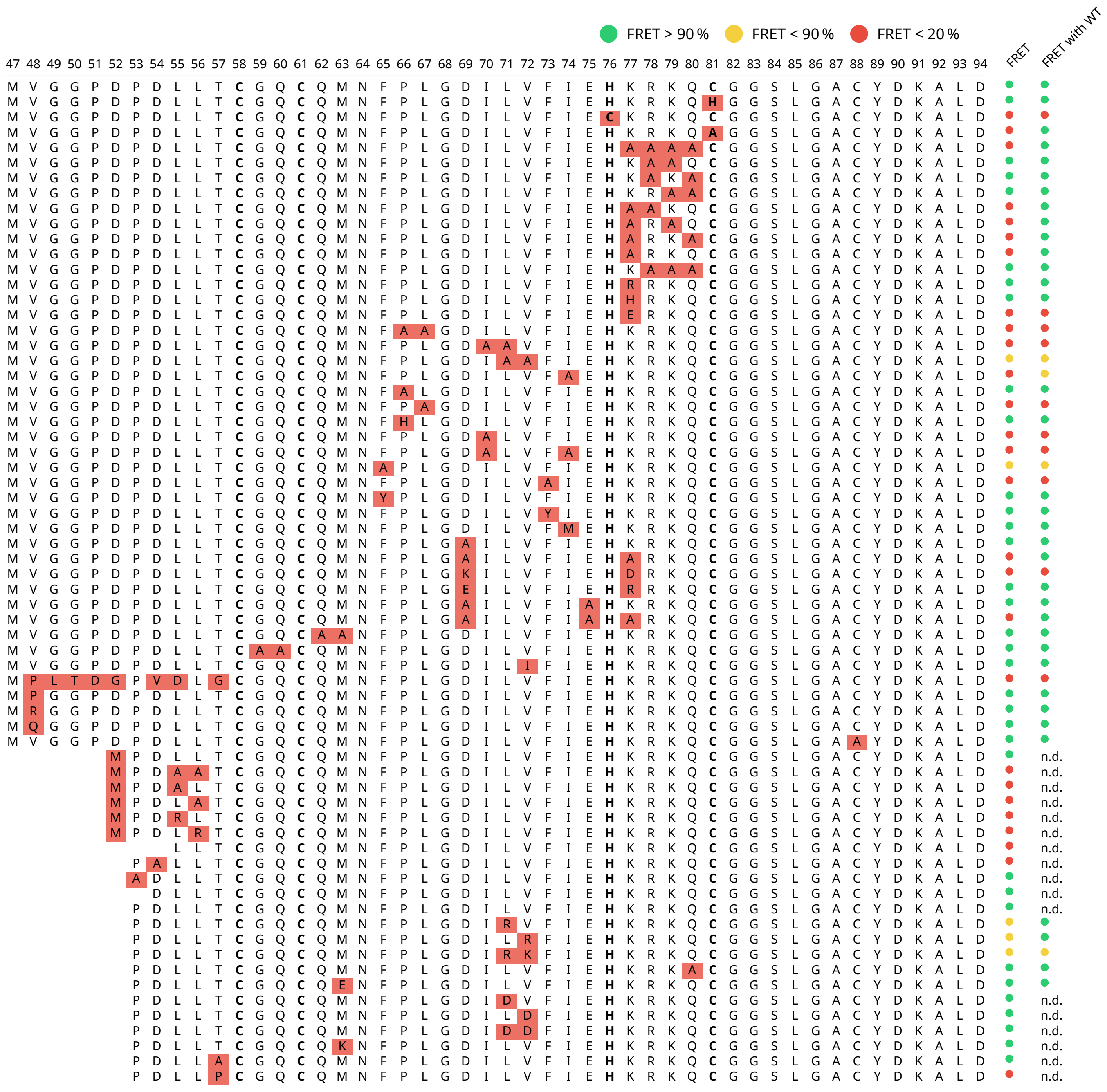

Figure S1: Full overview of all experimental residue mutations and their dimerization potential to itself and wild type.

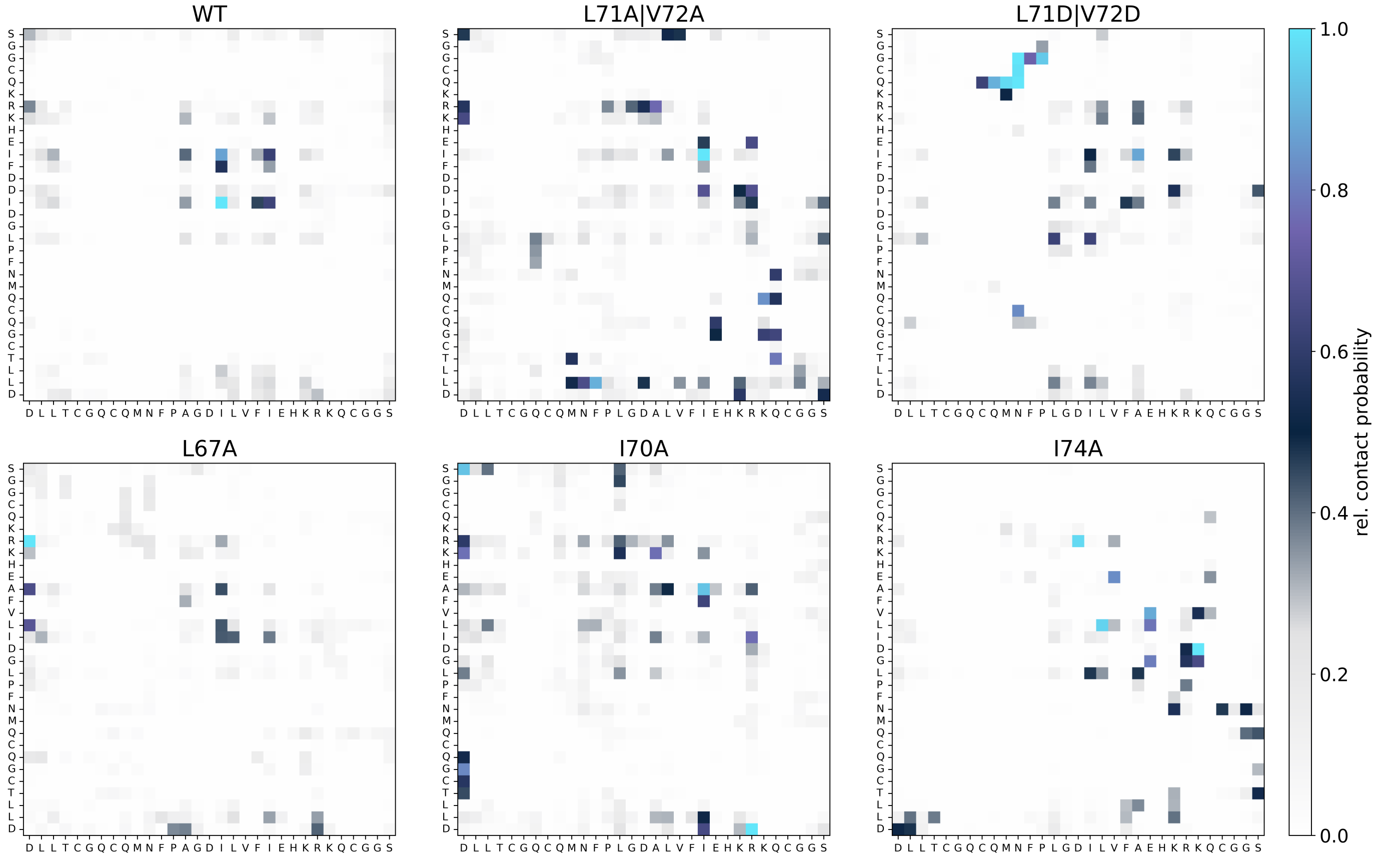

Figure S2: Relative probability of residue contacts during the TIGER2h protein-protein docking simulations for each mutant protein.
